# A Compendium for *Mycoplasma pneumoniae*

**DOI:** 10.3389/fmicb.2016.00513

**Published:** 2016-04-12

**Authors:** Gretchen L. Parrott, Takeshi Kinjo, Jiro Fujita

**Affiliations:** Department of Infectious Diseases, Respiratory and Digestive Medicine, Graduate School of Medicine, University of the RyukyusNishihara, Japan

**Keywords:** *Mycoplasma pneumoniae*, pneumonia, atypical pneumonia, community-acquired pneumonia, walking pneumonia, CARDS toxin

## Abstract

Historically, atypical pneumonia was a term used to describe an unusual presentation of pneumonia. Currently, it is used to describe the multitude of symptoms juxtaposing the classic symptoms found in cases of pneumococcal pneumonia. Specifically, atypical pneumonia is a syndrome resulting from a relatively common group of pathogens including *Chlamydophila* sp., and *Mycoplasma pneumoniae*. The incidence of *M. pneumoniae* pneumonia in adults is less than the burden experienced by children. Transmission rates among families indicate children may act as a reservoir and maintain contagiousness over a long period of time ranging from months to years. In adults, *M. pneumoniae* typically produces a mild, “walking” pneumonia and is considered to be one of the causes of persistent cough in patients. *M. pneumoniae* has also been shown to trigger the exacerbation of other lung diseases. It has been repeatedly detected in patients with bronchitis, asthma, chronic obstructive pulmonary disorder, and cystic fibrosis. Recent advances in technology allow for the rapid diagnosis of *M. pneumoniae* through the use of polymerase chain reaction or rapid antigen tests. With this, more effort has been afforded to identify the causative etiologic agent in all cases of pneumonia. However, previous practices, including the overprescribing of macrolide treatment in China and Japan, have created increased incidence of macrolide-resistant *M. pneumoniae*. Reports from these countries indicate that >85% of *M. pneumoniae* pneumonia pediatric cases are macrolide-resistant. Despite its extensively studied past, the smallest bacterial species still inspires some of the largest questions. The developments in microbiology, diagnostic features and techniques, epidemiology, treatment and vaccines, and upper respiratory conditions associated with *M. pneumoniae* in adult populations are included within this review.

## Introduction

*Mycoplasma pneumoniae*, was first discovered in Eaton et al. (1944). It was originally known as the Eaton agent. The tiny pathogenic agent could pass through a sterile filter, but could not be grown on standard bacteriologic media. Thus, during that time, it was originally thought to be a virus. Volunteer and field studies during the 1950s and early 1960s provided evidence verifying the Eaton agent was a cause of lower respiratory tract infections in humans (Chanock et al., 1960, 1961; Mufson et al., 1961). Chanock et al. (1962) was able to culture the Eaton agent on a cell-free medium and proposed both the taxonomic designation as well as the name of the organism as we know it today.

Currently, as one of the most studied mycoplasmas, a large collective knowledge on *M. pneumoniae* has accumulated. Recent discoveries in microbiology and improvements in diagnostic techniques and treatments have led to dramatic advances. At the same time knowledge regarding *M. pneumoniae* epidemiology, associated upper respiratory conditions, and vaccine candidates has also expanded. These topics and how they affect adult populations will be briefly covered in this review.

## Microbiology

With a small cell size and volume, just 1–2 μm long and 0.1–0.2 μm wide, mycoplasmas cannot individually be detected by light microscopy (Waites and Talkington, 2004) and colonies rarely exceed 100 μm in diameter. Sequenced in Himmelreich et al. (1996), the genome of *M. pneumoniae* was shown to consist of only 816,394 bp and 687 genes. Because of this small genome, the organism is limited in its capabilities and unable to synthesize rigid peptidoglycan cell walls. Alternatively, sterols provide the structural support in the triple-layer cell membrane. As a result, these organisms are insensitive to β-lactam antimicrobial agents, pleomorphic, and are unaffected by the gram staining method.

*Mycoplasma pneumoniae* reproduces via binary fission with well-organized chromosome segregation. Preceding binary cell fission, the attachment organelle, a specialized cellular structure that is responsible for cytadherence of this bacterium, duplicates (Krause and Balish, 2004; Balish, 2006; Balish and Krause, 2006). The resulting daughter organelle will migrate to the opposite pole of the cell before the completion of chromosomal separation. After duplication of chromosomal and cellular material, the now polar opposite attachment organelles will simultaneously bind to a surface and initiate gliding motility, pulling away from the central point; thus, creating daughter cells (Bredt, 1968; Miyata and Ogaki, 2006).

**FIGURE 1:**
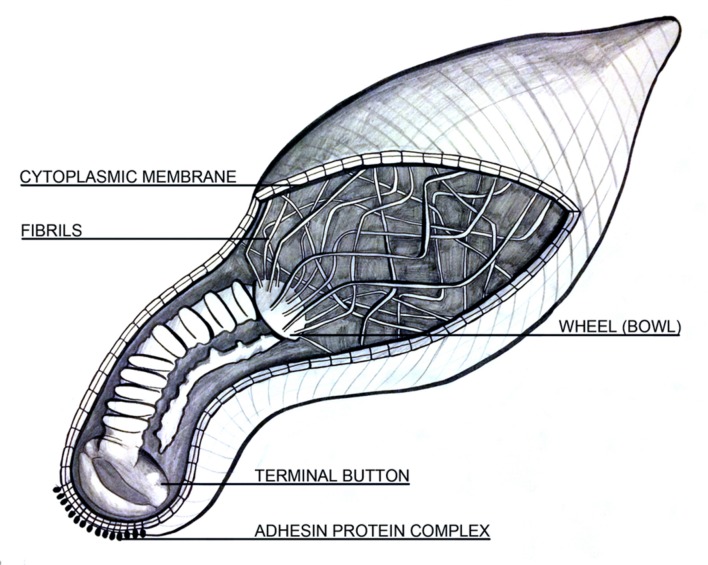
**A longitudinal schematic depicting the cellular architecture of *Mycoplasma pneumoniae.*** It contains known structural features including cell shape, lack of flagella, a terminal organelle including the “rod” composed of two segmented plates, one thick and one thin, and a wheel (bowl) complex with fibrils extending throughout the cytoplasm. The outer cell membrane is integrated with membrane proteins while the inner lining encloses the cytoplasm. Image is not to scale.

The attachment organelle is also crucial for host cellular interactions. Cytoskeletal proteins found within and around the attachment organelle facilitate adherence and motility. Of note, P1 and supporting proteins P30, P90, and P40 promote adhesion and binding to host cells via sialic acid receptors (Krivan et al., 1989; Roberts et al., 1989; Razin and Jacobs, 1992; Seto and Miyata, 2003; Waldo and Krause, 2006; Chaudhry et al., 2007), while P1, P30, P41, and P200 promote and regulate gliding motility (Kenri et al., 2004; Krause and Balish, 2004; Seto et al., 2005; Hasselbring and Krause, 2007; Jordan et al., 2007). Many authors allude to a cellular construction based on electron micrograph observations of cryosections (Hegermann et al., 2002; Henderson and Jensen, 2006; Seybert et al., 2006; Nakane et al., 2015). Integration and interpretation of these studies propose that the attachment organelle is connected to a rod consisting of two paired plates (rods), one thick and one thin, which is attached, at the proximal end, to a wheel-like protein complex (bowl-complex), the wheel (bowl) further connects to other cytoskeleton filaments in the peripheral cell. A schematic, assembled from literature interpretation, is depicted in **Figure 1** (Meng and Pfister, 1980; Razin et al., 1998; Trachtenberg, 1998; Krause and Balish, 2001, 2004; Hegermann et al., 2002; Henderson and Jensen, 2006; Miyata and Ogaki, 2006; Seybert et al., 2006; Nakane et al., 2015). However, the exact mechanism by which the attachment organelle and associated structures and proteins initiate motility is still relatively unknown, although numerous authors support inchworm-like movement (Henderson and Jensen, 2006; Hatchel and Balish, 2008; Nakane et al., 2015).

*Mycoplasma pneumoniae* has not been found freely living in nature. It is predominantly considered a mucosal pathogen existing parasitically on the epithelial surface of its host. Interactions between *M. pneumoniae* and host tissue, continuously show the attachment organelle bound to the host cell surface (Wilson and Collier, 1976; Razin and Jacobs, 1992; Rottem, 2003). However, reports of internalization by lung epithelial cells also exist (Yavlovich et al., 2004). It is assumed cytadherence protects mycoplasma from clearance via the mucociliary apparatus and this attachment is ultimately considered the initiating event of disease.

## Pathogenesis

Once bound to host tissue, pathogenic processes begin to occur. Hydrogen peroxide and superoxide, produced by *M. pneumoniae* through the metabolism of glycerol, have been shown to cause injury to epithelial cells and their associated cilia (Somerson et al., 1965; Low, 1971; Tryon and Baseman, 1987; Minion and Jarvill-Taylor, 1994). The effects of hydrogen peroxide on host cells such as erythrocytes include denaturation of hemoglobin, peroxidation of lipids, and eventual cell lysis. The same oxidative stress in the respiratory epithelium can result in both structural and functional deterioration of cilia (Waites and Talkington, 2004).

Recently discovered, the community-acquired respiratory distress syndrome (CARDS) toxin has also been shown to facilitate localized disruption and cytotoxicity (Kannan et al., 2005, 2010; Kannan and Baseman, 2006; Johnson et al., 2011; Becker et al., 2015). Cells exposed to this 68-kDa proteinaceous toxin, with homologies to the pertussis toxin, exhibited distinct vacuolization and cell rounding (Kannan and Baseman, 2006). Other cellular effects of CARDS could include the loss of cilia, reduced oxygen consumption, glucose utilization, and amino acid uptake, as well as detachment and ultimate shedding of the infected cells, documented elsewhere (Clyde, 1971, 1983; Collier, 1983; Johnson et al., 2011). The dry, hacking cough commonly associated with early infection, is most likely the clinical manifestation of the aforementioned cellular damages endured by the upper respiratory tract (Waites and Talkington, 2004).

The CARDS toxin has also been shown to activate its own pathogenic response in animal models. A direct relationship between the number of *M. pneumoniae* organisms, amounts of the CARDS toxin, and severity of lung histopathology was observed in murine models (Techasaensiri et al., 2010; Kannan et al., 2011). Moreover, mice and baboons, which received recombinant CARDS toxin alone, elicited cellular inflammation similar to *M. pneumoniae* infection. This suggests the CARDS toxin, plays a major role in pathogenesis (Hardy et al., 2009).

**FIGURE 2:**
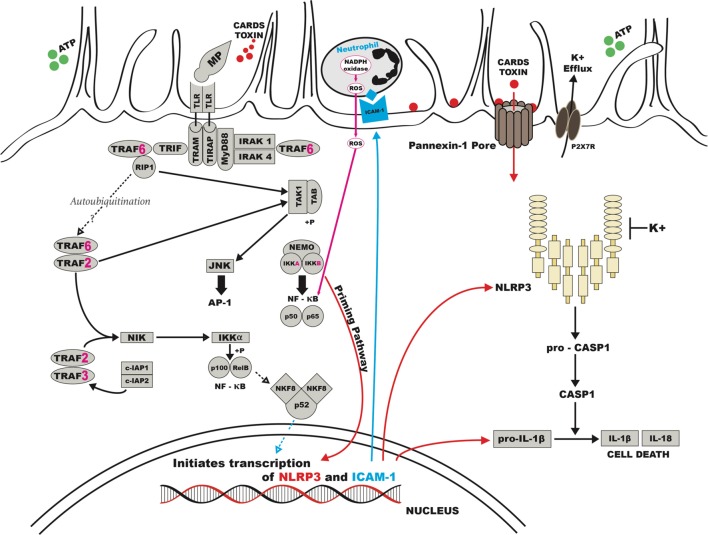
**Possible schematic for pathogenesis of human M. *pneumoniae*.** CARDS, Community Acquired Respiratory Distress Syndrome; ATP, adenosine tri-phosphate; K, potassium; ICAM, intracellular adhesion molecule; NADPH, nicotinamide adenine dinucleotide phosphate; ROS, reactive oxygen species; TLR, toll like receptor.

Further, research has shown the CARDS toxin can localize with the NOD-like receptor containing pyrin domain 3 (NLRP3) inflammasome and catalyze the ADP-ribosylation of NLRP3 (Bose et al., 2014). As a result, the CARDS toxin is the first example of a toxin exhibiting both ADP-ribosylating and vacuolating properties (Krueger and Barbieri, 1995; Kannan and Baseman, 2006). In **Figure 2**, we propose a potential cell signaling model based on information collected from previously published reports (Fan et al., 2003; Yang et al., 2004; Chu et al., 2005; Xiang and Fan, 2010; Johnson et al., 2011; Kannan et al., 2011; Franchi and Nunez, 2012; Franchi et al., 2012; Bose et al., 2014; Saraya et al., 2014; Shimizu et al., 2014; Vanaja et al., 2015).

Interaction between respiratory epithelial cells and surface lipoproteins of *M. pneumoniae* is likely to induce the host immune system via Toll-like receptor (TLR)-2 (Chu et al., 2005; Kraft et al., 2008) or TLR-4 (Shimizu et al., 2014) stimulating the synthesis of intracellular adhesion molecule (ICAM) receptors. Stimulation and crosstalk of the TLRs can trigger and amplify the production of chemokines promoting lymphocyte and neutrophil trafficking and inflammation in the lung (Fan et al., 2003; Yang et al., 2004; Saraya et al., 2011). In addition to respiratory epithelial cells, *M. pneumoniae* has been shown to directly activate and induce the production of cytokines from unsorted peripheral blood leukocytes (Kita et al., 1992), lymphocytes (Simecka et al., 1993; Medina et al., 2012), and monocytes and macrophages (Yang et al., 2003; Broaders et al., 2006). Opsonization may also occur but the evidence is minimal since re-infection is common.

Macrophages, including alveolar macrophages likely play a central role as an innate immune defense mechanism through phagocytosis. Alveolar macrophages, in particular, can also secrete pro-inflammatory cytokines, such as RANTES, which is a known chemo-attractant for neutrophils and basophils (Bischoff et al., 1993; Saraya et al., 2011; Tani et al., 2011). According to some reports, the most distinguishing pathological feature resulting from this organism in human pneumonia is an increase of plasma cell-rich lymphocytic infiltration in the peri-bronchovascular areas (PBVAs), with accumulation of macrophages, neutrophils, and lymphocytes in alveolar spaces (Coultas et al., 1986; Hayashi et al., 1986; Rollins et al., 1986; Saraya et al., 2014).

The underlying cell-mediated immunity of the host also plays an important role in the progression and development of *M. pneumoniae* related diseases. Furthermore, cell-mediated immunity level or predominant response is potentially correlated to the variable pulmonary patterns seen in chest images (Tanaka et al., 1996; Saraya et al., 2011, 2014). Multiple studies have acknowledged the importance of IL-12, interferon-γ, and Th1 type T-cell responses during the course of *M. pneumoniae* infections (Fonseca-Aten et al., 2005; Tagliabue et al., 2008; Hardy et al., 2009; Techasaensiri et al., 2010). However, a recombinant CARDS toxin has resulted in potent allergic-type pulmonary inflammation characterized by T-cell dependence, airway hyperreactivity, and production of Th2 type cytokines (Medina et al., 2012). In sensitized mice, *M. pneumoniae* can lead to Th2 type T-cell allergic inflammation (Chu et al., 2003, 2005). While, other previous animal studies have shown the histopathological score of *M. pneumoniae* pneumonia is significantly higher in BALB/c mice (Th2 predominant) than in C57BL/6 mice (Th1 predominant; Fonseca-Aten et al., 2005). The collective interpretation of all these findings suggest an attractive link to the differences observed in human host cell-mediated immunity response against the organism and the resulting variable pathogenic patterns seen in chest images and serological responses (Kraft et al., 1998; Nisar et al., 2007; Atkinson et al., 2009; Saraya et al., 2011).

## Epidemiology

*Mycoplasma pneumoniae* is able to infect both the upper and lower respiratory tracts and it can create both endemic and epidemic situations among children and adults worldwide. From 2001 to 2006, *M. pneumoniae* was the most common atypical pathogen identified in 39 hospitals across 11 countries (Arnold et al., 2007). However, most available data regarding *M. pneumoniae* infections comes from studies performed in Japan, Europe, and the United States. Within Europe*, M. pneumoniae* is frequently included in regular surveillance, but studies from arctic and tropical zones point to the many diverse populations with infections due to this organism (Suhs and Feldman, 1966; Golubjatnikov et al., 1975; Joosting et al., 1976; Campos et al., 1993).

*Mycoplasma pneumoniae* has a diminutive size, which allows it to spread from person to person through droplet infection during close contact. Following a coughing event, contaminated droplets disperse through the air. The eventual cytadherence by the bacteria in an alternate host describes successful transmission. Some patients may remain infectious for prolonged periods despite the disappearance of many symptoms, other than cough (Hallander et al., 1999; Wadowsky et al., 2002; Ishida et al., 2010; Wang et al., 2011).

Immunity is not long lasting; the bacteria and its associated disease can relapse in patients even after adherence to an effective antibiotic regimen (Watson and Storch, 2008). The genomic variation of the P1 adhesin may contribute to this complex and recurring epidemiology. There are two main subtypes of *M. pneumoniae* frequently isolated from clinical specimens (Su et al., 1990), though other variants have been reported (Dorigo-Zetsma et al., 2000; Dumke et al., 2010b; Pereyre et al., 2012). Japan has reported the cycling of prevalent subtypes. Between 1995 and 2001, subtype 2 was accountable for the majority of infections, but between 2002 and 2005, subtype 1 became more prevalent (Kenri et al., 2008). It is not yet understood if recurrence within one individual is caused by reactivated infection or exposure to different genetic subtypes.

Interestingly, epidemic and endemic settings also report a polyclonal spread of the bacteria (Chalker et al., 2011; Pereyre et al., 2012, 2013), with multiple types or strains propagating within the human population simultaneously. This observation indicates point source infection is not the probable cause of countrywide epidemics; rather, a more likely cause is some environmental factor. Rates of *M. pneumoniae* vary annually, yet cyclic epidemic patterns have been observed every three to five years in long-term studies and geographic surveillance (Lind et al., 1997; Nir-Paz et al., 2012). It has been suggested most epidemics occur in either late summer or autumn within North America (Alexander et al., 1966; Feikin et al., 1999). However, other geographic regions report maintained epidemics through all seasons (Foy et al., 1979; Blystad et al., 2012; Nir-Paz et al., 2012; Polkowska et al., 2012; Uldum et al., 2012). A recent study from Fukuoka, Japan has reported climactic events related to the El Niño Southern Oscillation and Indian Ocean Dipole were significantly associated with monthly incidence of *M. pneumoniae* in both 2005–2007 and 2010–2011 (Onozuka and Chaves, 2014). These weather events may be responsible for both the cyclic 3–5 years resurgence pattern as well as the differing seasonality across continents.

In adults, *M. pneumoniae* is potentially responsible for more than 35% of hospitalized community-acquired pneumonia (CAP) cases (Marston et al., 1997; Dey et al., 2000; Cunha and Pherez, 2009). However, a recent report from the Centers for Disease Control and Prevention, estimated only 2% of detectable pathogens in hospitalized CAP patients were due to *M. pneumoniae* (Jain et al., 2015). Moreover, a report from the Atypical Pathogens Reference Laboratory Database attributes 12% (range 11–15%) of global CAP incidence to *M. pneumoniae* (Arnold et al., 2007). This last estimate does take into account the fluctuation of both seasonal and outbreak years, covering almost five winter seasons in its data analysis. However, patients with CAP typically have mild symptoms and are treated as outpatients, if at all. Therefore, the number of *M. pneumoniae* cases reported may be an underestimation of actual burden.

Prevalence, documented in other studies, can have a wide range Marston et al. (1997), reported 5.4% of CAP in hospitalized adults in the United States was due to *M. pneumoniae* by serology. Also using serology in Porath et al. (1997), from Israel, reported 29.2% of hospitalized CAP adults were infected with *M. pneumoniae*. Wattanathum et al. (2003), Thailand reported similarly high rates by serology, with 29.6% of outpatients with symptoms due to *M. pneumoniae.* Whereas, von Baum et al. (2009), polymerase chain reaction (PCR) and serological analysis from Germany showed 6.8% of CAP cases were due to *M. pneumoniae*, of which 55% were treated as outpatients. Prevalence reporting for most countries, however, is difficult due to the non-availability of reliable, rapid diagnostic techniques and an organized reporting system (Kashyap and Sarkar, 2010).

**FIGURE 3:**
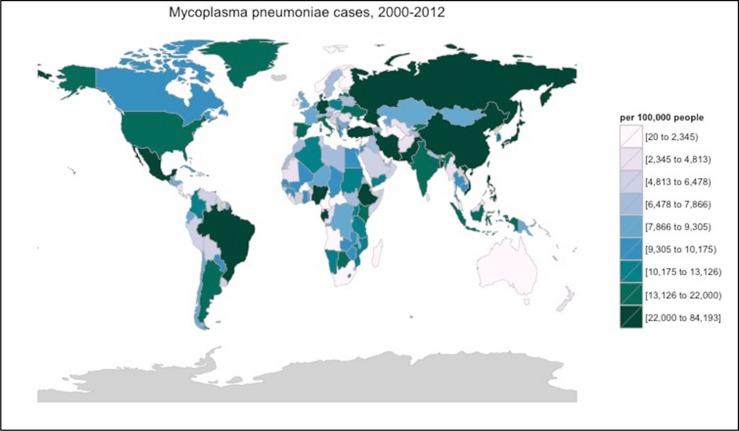
**Interpolated *M. pneumoniae* incidence from 2000 to 2012.** These statistics were calculated using linear interpolation from reported incidence found within the literature. Gray countries were incalculable. This interpolation process did not take into account any genetic, cultural, environmental, social, or other differences across the various countries and regions. Thus, interpolations may have very limited relevance to the actual incidence of *M. pneumoniae* in any region. Image created using R v.3.2.2 with the package choroplethr.

We calculated an estimated number of *M. pneumoniae* cases per 100,000 people by linear interpolation based on in country reports collected during our review process (Bii et al., 2002; Chaoprasong et al., 2002; Accomando et al., 2004; Nagalingam et al., 2004; Obeidat et al., 2005; Matute et al., 2006; Petitjean Lecherbonnier et al., 2006; Shankar et al., 2006; Somer et al., 2006; Kung, 2007; Huang et al., 2008; Boettcher et al., 2010; Prodromidou et al., 2010; Touati et al., 2010; Eick et al., 2011; Song et al., 2011; Wang et al., 2011; Bajraktarevic et al., 2012; Blystad et al., 2012; Feikin et al., 2012; Hoffmann et al., 2012; Lenglet et al., 2012; Polkowska et al., 2012; Uldum et al., 2012; Wellinghausen et al., 2012; Chen et al., 2013, 2015; Hong et al., 2013; Kawai et al., 2013; Luchsinger et al., 2013; Wu et al., 2013; Carrim et al., 2014; Grassi et al., 2014; Moore et al., 2014; Neocleous et al., 2014; Zhao et al., 2014; Kogoj et al., 2015; Liu et al., 2015). The resulting data is shown in **Figure 3**, with elevated rates possibly found throughout China, Russia, Mexico, and Brazil.

The majority of outbreaks have occurred within a community or in closed or semi-closed settings such as military bases or universities (Mogabgab, 1968; Edwards et al., 1976; Gray et al., 1997, 1999; Feikin et al., 1999; Crum et al., 2005; Walter et al., 2008; Centers for Disease Control and Prevention [CDC], 2012, 2013; Waller et al., 2014), hospitals (Fischman et al., 1978; Kashiwagi et al., 1985; Kleemola and Jokinen, 1992; Hyde et al., 2001; Shangguan et al., 2014), and facilities for the developmentally disabled or elderly (Marrie, 1993; Klausner et al., 1998; Hastings et al., 2015). Although, these outbreaks can disrupt and consume significant resources in the workforce, long-term morbidity is uncommon (Waites and Talkington, 2004). Controlling an outbreak often includes simple strategies such as, cohorting infected patients and the correct use of antibiotics as treatment.

## Clinical Manifestations

In the acute phase of infection, a dry cough develops which may progress to a wet cough in 3–4 days. Coughing represents progressing tracheobronchitis, the most common form of infection. Chest auscultation may be unhelpful for diagnosis in most situations, but scattered rhonchi and expiratory wheezes may sometimes present (Norisue et al., 2008). If pneumonia develops, atypical pneumonia is the predominant syndrome observed in adult patients. The syndrome is portrayed by the gradual onset of pharyngitis, sinus congestion, infrequent otitis media, and eventually prolonged lower respiratory involvement up to and including pneumonia with low-grade fever and bibasilar pulmonary infiltrates. The incubation period prior to symptom emergence may be short or as long as 3 weeks. In severe pneumonia cases, dry rales and frank consolidation may be observed, but this is fairly uncommon, and may be due to co-infection with *Streptococcus pneumoniae* or *Chlamydophila pneumoniae* (Nambu et al., 2006; Norisue et al., 2008).

There may be mild leukocytosis, but the total white blood cell count does not often exceed 15,000/μL. Expectorated sputum is not viscous. If sputum is sufficient, gram staining shows nothing discernable due to the small size of *M. pneumoniae* and its lack of cell wall. Severe cough and chest images depicting bronchopneumonia are common, as inflammation occurs in response to ciliated cell damage. Respiratory symptoms in severe pneumonia cases may necessitate admission to the hospital due to decreased blood oxygen and increasingly labored breathing. The most common radiological and high resolution computed tomography (CT) images of *M. pneumoniae* pneumonia include air-space opacification, bronchovascular thickening, atelectasis, nodular infiltration, and linear opacities outward from the hilum, but are indistinguishable from other bacterial or viral pneumonia patterns (Reittner et al., 2000; Miyashita et al., 2009). Putman et al. (1975) proposed three possible reasons for alternate chest images: the existence of an underlying or co-existing pulmonary disease, previous exposure to *M. pneumoniae*, or the varied immune response and host susceptibility. To date, many researchers favor prior exposure (Saraya et al., 2011; Medina et al., 2012) and host immune differences (Tanaka et al., 1996; Saraya et al., 2014). Still, others report alternate host differences such as age (Lee et al., 2006), health conditions (Lambert, 1968), or environmental factors (Putman et al., 1975; Yang et al., 2004). Severity of disease has also been shown to be strain- and toxin concentration-dependent (Techasaensiri et al., 2010).

As mentioned previously, the cell-mediated immunity of patients may have a strong impact on the course of disease development following *M. pneumoniae* infection (Putman et al., 1975; Tanaka et al., 1996; Yang et al., 2002; Saraya et al., 2011, 2014). Some studies report detection of *M. pneumoniae* in seemingly healthy individuals. One such study reports peak incidence of 13.5% of 758 healthy volunteers (Gnarpe et al., 1992), while another study detected positive throat cultures 4 months after illness (Foy et al., 1966). It is possible these asymptomatic or mild infections still allow for shedding of the pathogen. These patients may act as a reservoir from which further spreading can occur. Similar to other respiratory infections, the duration of signs and symptoms will be shorter if antibiotics are initiated early in the course of infection.

### Other Respiratory Manifestations

It has been suggested *M. pneumoniae* infection contributes to the development of chronic respiratory diseases, including persistent cough and asthma. While medical care for a persistent cough is frequently sought out, this symptom is commonly associated with *M. pneumoniae* in children (Hallander et al., 1999; Wang et al., 2011) and older adults (Miyashita et al., 2008; Takahashi et al., 2009). The organism may be found frequently in school-aged children with persistent cough particularly during active epidemics of *M. pneumoniae* (Wang et al., 2011) or concurrently with *Bordetella pertussis* (Hallander et al., 1999). Similar outbreaks of persistent cough are likely to occur in other ideal settings, such as dormitories, among military recruits, and in hospitals or nursing homes. In Japan, *M. pneumoniae* was confirmed by serology in 5.5% of adult patients with persistent cough (Ishida et al., 2010). Despite this evidence, Wadowsky et al. (2002), concludes *M. pneumoniae* is infrequently the active agent of cough illnesses longer than 5 days in adolescents and adults. However, cough may persist after acute infection in adults due to the continued presence of mycoplasma cell products or the CARDS toxin (Kannan et al., 2011).

*Mycoplasma pneumoniae* has for a long time been implicated in the exacerbation of asthma (Biscardi et al., 2004; Nisar et al., 2007; Hong, 2012; Wood et al., 2013). Moreover, some studies have isolated the bacteria in higher prevalence among asthmatics (Kraft et al., 1998; Smith-Norowitz et al., 2013). Sutherland et al. (2004), evaluated a questionnaire given to patients with a history of CAP after an episode of pneumonia. There, patients with a history of atypical pneumonia were more likely to be asthmatics. Other studies go on to document *M. pneumoniae* infection preceding an initial asthmatic event (Mok et al., 1979; Yano et al., 1994; Biscardi et al., 2004). It is possible mycoplasma infection leads to the destruction of respiratory cells and facilitates mucosal penetration by other antigens. Thereby, allowing the patient to become atopic to *M. pneumoniae* and other allergens (Nisar et al., 2007). However, at this time, none of these studies have distinguished increased susceptibility or exposure to *M. pneumoniae* from genetic predisposition for asthma (Mok et al., 1979).

Although, the *M. pneumoniae* connection with asthma is well-established, the mechanism behind development of the disease is still relatively unknown. One clue to pathogenesis in this regard may be immunoglobin (Ig) E responses. Some studies have reported control patients, may be capable of mounting a higher antibody response than those with asthma (Kraft et al., 2002; Atkinson et al., 2009; Wood et al., 2013). The role of T lymphocytes in the pathogenesis of asthma has been well-documented. The release of type 2 cytokines, including interleukin (IL)-4 and 5, is also increased in the serum of patients with *M. pneumoniae* (Esposito et al., 2002; Jeong et al., 2012). These cytokines in turn promote IgE production, which also plays a part in asthma. Also, antigenic mycoplasmas may initiate an antibody response resulting in IgE attaching to mast cells interacting with *M. pneumoniae*, which ultimately stimulates histamine release (Gil et al., 1993). However, additional studies are needed to fully understand the role of *M. pneumoniae* plays in the initial onset and exacerbation of asthma.

### Extrapulmonary Manifestations

Extrapulmonary manifestations, although less common, have also been described. Patients with compromised immunity, including humoral immunodeficiences may be at higher risk for developing these complications. Extrapulmonary complications may occur in no more than 10% of patients with *M. pneumoniae*. Central nervous system (CNS) complications comprise the bulk of commonly seen extrapulmonary manifestations (Guleria et al., 2005). Such complications include, encephalitis, meningitis, optic neuritis, and Guillain–Barré syndrome among others. The mechanism of action behind these ominous manifestations remains unknown. In most patients, respiratory illness precedes, 2–14 days before, CNS findings (Tsiodras et al., 2005). CNS complications may result from direct invasion of *M. pneumoniae* in the brain (Tsiodras et al., 2005) or through extreme immune-mediated damages (Lee et al., 2013). Immune-mediated responses could be the result of cross-reacting antibodies and antigens shared by *M. pneumoniae* and the brain, depression of T-lymphocyte function, immune complex deposition, or intravascular clotting (Guleria et al., 2005; Tsiodras et al., 2005; Johnson, 2006).

Dermatological conditions such as erythematous maculopapular, vesicular rashes and Stevens-Johnson syndrome, are also somewhat common as extrapulmonary manifestations (Walicka et al., 2008; Kashyap and Sarkar, 2010; Kunimi et al., 2011; Shimizu et al., 2012). Whereas, hematological, gastrointestinal, musculoskeletal, renal and other inflammatory manifestations have occurred in rare cases (Cameron et al., 1992; Perez et al., 1997; Parisi and Filice, 2001; Perez and Artola, 2001; Waites and Talkington, 2004; Johnson et al., 2007; Atkinson et al., 2008; Maia et al., 2009; Kashyap and Sarkar, 2010; Bayram et al., 2011). Further, information regarding the details of extrapulmonary diseases as a result of *M. pneumoniae* can be found in numerous case reports. However, because *M. pneumoniae* is quite common, there is the possibility some of these instances are only coincidental.

## Diagnosis

As a common cause of illness for both children and adults*, M. pneumoniae* should regularly be considered as a possible etiology in any upper respiratory infection, especially in immunocompromised patients or patients who have not responded to β-lactam antibiotics. Recently, there are many new techniques adapted to detecting the presence of *M. pneumoniae* suitable for both research and diagnostic purposes. These techniques were described in length by Daxboeck et al. (2003). Here, we will include a brief review and updated techniques.

The Japanese Respiratory Society (JRS) developed a scoring system to differentiate between typical and atypical pneumonia (Ishida et al., 2007) using clinical findings. The differential items include: (1) patient under 60 years of age; (2) no or minor underlying diseases; (3) stubborn cough; (4) poor chest auscultatory findings; (5) no sputum or etiologic agent identified by gram staining; and (6) a peripheral white blood cell below 10,000/μL. In cases where a patient presents with four or more of the six items, JRS guidelines recommend the use of macrolides or tetracyclines because of a suspected atypical pneumonia.

Many countries consider the most reliable diagnosis for acute pneumonia infection would come from a combination of two or more separate laboratory methods, such as serology and PCR (Petitjean et al., 2002; Daxboeck et al., 2003; Martinez et al., 2008; Nilsson et al., 2008; Chaudhry et al., 2013), or a clinical prediction rule, such as the JRS scoring system, with a rapid laboratory test (Ishida et al., 2007; Miyashita et al., 2011, 2015). The use of the laboratory tests listed below along with clinical prediction rules can more easily distinguish among acute, persistent infection and asymptomatic patients.

Historically, the use of cold agglutinins and detection of *M. pneumoniae* by culture methods were widespread diagnostic techniques. Cold agglutinin testing was once considered a valuable tool, but it is not a highly specific indicator of *M. pneumoniae*, as autoantibodies in the blood can be elevated from other diseases or syndromes (Jacobs, 1993; Beersma et al., 2005). A decade ago, the complement fixation (CF) method that detects the human body’s early responses to *M. pneumoniae* was common around the world. A single 1:64 CF titer was considered an indication of recent *M. pneumoniae* infection. However, the CF test has a well-documented lack of sensitivity and specificity (Ponka et al., 1981; Waites and Talkington, 2004).

### Rapid Diagnostics

Currently, a more advanced test using a similar method to CF is the microparticle agglutination assay (MAG), wherein specific antibodies to *M. pneumoniae* create hemagglutination and erythrocytes are replaced by latex particles to avoid non-specific reactions (Barker et al., 1990). Additionally, in August 2013, two rapid antigen kits for the detection of *M. pneumoniae* in nasopharyngeal samples became available in Japan (Miyashita et al., 2015; Yamazaki et al., 2015). These rapid antigen kits detect two different targets L7/L12 ribosomal protein or P1 adhesion protein. Two studies have compared the ribosomal protein rapid antigen kit to real-time PCR and the resulting theoretical diagnostic sensitivities were approximately 60% (Miyashita et al., 2015) and 74% (Yamazaki et al., 2015) in these samples. Hatano et al. (2013), reported the use of this rapid P1 adhesion protein detecting kit in 462 patients with a resulting sensitivity and specificity of 90 and 89.5%, respectively. However, further prospective studies are needed to evaluate the sensitivity of these rapid tests more thoroughly.

Rapid diagnostic tests are most useful in the early stages of CAP to assist decisions related to patient therapy. Despite the development of these two tests, however, the majority of *M. pneumoniae* cases continue to be diagnosed using serological methods or through the detection of nucleic acids. A variety of tests have been developed in this regard, each with their own advantages and disadvantages. However, numerous confounding variables inherent to the pathogenesis of *M. pneumoniae* contribute to obscuring the diagnostic accuracy of laboratory methods.

### Serology

Serology remains as relevant now as it was in the past for the diagnosis of *M. pneumoniae.* The ease of sample collection coupled with further regard to conclusive evidence of *M. pneumoniae* as the causative agent, maintain serology’s presence within diagnostics. There are currently several commercially available serological tests utilizing a variety of methods to detect the presence of *M. pneumoniae.*

Enzyme immunoassays (EIAs) use whole-cell lysates, containing glycolipid antigens, or protein extracts without glycolipid antigens. IgG seroconversion in *M. pneumoniae* infected patients is estimated to occur from 3 to 8 weeks following infection. EIAs are more sensitive than both the CF and MAG tests (Moule et al., 1987; Aubert et al., 1992; Nir-Paz et al., 2006) for detecting acute infection. Most EIAs implement a 96 well-microtiter plate. However, rapid membrane based procedures are available for the detection of a single specimen (Alexander et al., 1996; Matas et al., 1998).

For diagnosis during acute infection, a separate detection of IgM or IgA is useful. IgM antibodies appear in the first week of illness and reach their highest titers during the third week (Jacobs, 1993). IgA antibodies are also produced in early stages of the disease (Watkins-Riedel et al., 2001). Assays for IgM and IgA detection are primarily based on the enzyme-linked immunosorbent assay (ELISA) principle. Rapid assays, like those previously mentioned are also available and generally use direct immunoflourescence, counter immunoelectrophoresis, immunoblotting or antigen capture EIs (Kashyap and Sarkar, 2010). Talkington et al. (2004) and Beersma et al. (2005), more thoroughly evaluated commercially available EIAs.

Despite its many strengths and versatility, serology lacks sensitivity. Many ELISA tests have the possibility of false positive results by cross-reactions with other mycoplasma species (Morrison-Plummer et al., 1987). Additionally, antibodies to *M. pneumoniae* may not appear until 2 weeks following initial infection and onset of symptoms (Vikerfors et al., 1988). Other studies report substantially longer times until positive serology results (Nir-Paz et al., 2006; Nilsson et al., 2008; Zhang et al., 2011). Further still, physicians must also consider the status of a patient’s immune system. Particularly, in adults the response to IgM may be non-specific or absent (Uldum et al., 1992); while other underlying conditions may indicate an immunocompromised patient. However, serology as a diagnostic approach remains a convenient alternative for the detection of *M. pneumoniae* in respiratory secretions.

### Nucleic Acid Amplification

Polymerase chain reaction amplification from respiratory secretions, such as nasopharyngeal, oropharyngeal, or sputum samples can provide more sensitive detection. Many studies have shown amplification methods can detect *M. pneumoniae* even in seemingly healthy individuals (Leng et al., 1994; Tjhie et al., 1997). PCR tests have been designed around the 16S rDNA, P1 adhesion protein, and the ATPase operon genes of *M. pneumoniae.* Sensitivity can be further increased by nested PCR, which involves reamplification of a PCR product with a different set of primers for the same target.

Quantitative real-time PCR (qRT-PCR) may also provide an attractive alternative to serology and conventional PCR (Pitcher et al., 2006). It is as sensitive as conventional PCR with the additional possibility of quantitative capabilities, which may indicate acute infections. The qRT-PCR method has also been successful for rapidly and reliably distinguishing between the two dominant *M. pneumoniae* types (Schwartz et al., 2009). Furthermore, a qRT-PCR assay, designed to target the CARDS toxin gene, proved to be the most sensitive assay to identify positive specimens in an outbreak investigation and again in other specimens (respiratory and cerebrospinal fluid) in sporadic cases (Winchell et al., 2008). Currently, a commercially available kit, implementing detection of the CARDS toxin gene does not exist. A publication authored by Chaudhry et al. (2013) indicates in-house qRT-PCR designed methods have a small but significant increase in sensitivity over traditional PCR methods, but primer design and standardization could be problematic for less experienced laboratories.

The use of conventional PCR and qRT-PCR has standardized the detection of *M. pneumoniae* along with other pneumonia and atypical pneumonia inducing bacterial pathogens (Mustafa et al., 2011). A recent study comparing four commercially available multiplex PCR assays found performance across different manufacturers remains relatively high and stable, with 93–100% agreement for all comparisons (Anderson et al., 2013). Khanna et al. (2005) developed a multiplex PCR assay for detection of five pneumonia-causing bacteria; it is now available commercially. The use of multiplex PCR or multiplex qRT-PCR technology enables the detection of multiple pathogens simultaneously with excellent sensitivity and specificity. Multiplex technology is particularly useful for the diagnosis of CAP patients, in etiological studies, or when broad-spectrum antibiotics fail to improve patient conditions.

Developed in Japan, a new amplification technique called loop-mediated isothermal amplification (LAMP) has also been applied to rapid diagnosis of *M. pneumoniae* (Saito et al., 2005; Yoshino et al., 2008; Kakuya et al., 2014). This molecular amplification method occurs in a single tube at constant temperature, eliminating the necessity of a thermocycler. Endpoint analysis can be performed rapidly by visual confirmation of turbidity and precipitates or can be integrated into more advanced photometrics for more accurate quantification. The sensitivity of the LAMP assay was 88.5% compared to a validated qRT-PCR test on samples collected in the United States, and no cross reactivity was observed against 17 other mycoplasma species, human DNA, nor other common respiratory pathogens (Petrone et al., 2015). Unfortunately, due to the limits inherent within primer design, multiplexing of this assay is not possible. In the near future LAMP assays could enable rapid, low cost detection of *M. pneumoniae* cases and earlier recognition of outbreaks by medical providers (Petrone et al., 2015), particularly in resource limited settings.

### Culture Techniques

Although still seen as the “gold standard,” bacterial culture for *M. pneumoniae* from oropharyngeal samples can be time consuming due to the nutritive requirements. Specificity is 100%, when protocols are successful. Isolation of the pathogen has advanced the knowledge surrounding extrapulmonary manifestations, because successful isolation provides evidence of direct invasion by living bacteria (Daxboeck et al., 2003). Similar conclusions cannot be made from all positive PCR results, because target DNA may still be detected in patients beyond the death of the bacteria. However, the sensitivity of culture for diagnosis can be low and dependent both on the skill of the laboratory as well as the quality of the specimen. Thus, *M. pneumoniae* is cultured with cell-free media formulations, primarily for research purposes.

## Chemotherapy and Vaccines

These organisms lack a peptidoglycan cell wall, therefore therapy, which interferes with DNA synthesis, i.e., quinolones, or protein synthesis such as, macrolides and tetracyclines, are used more frequently than beta-lactams and glycopeptides and generally have a greater influence on disease (Niederman, 2001; Watkins and Lemonovich, 2011). For young children, macrolides should be considered first, due to potential severe side effects of tetracyclines and quinolones (Suzuki et al., 2006).

Macrolides are well-known for their antibiotic capabilities. However, considerable data has also been gathered confirming macrolides also possess anti-inflammatory properties, which can also contribute to patient improvement (Gotfried, 2004; Tamaoki, 2004). Macrolides seem to modify or regulate the immune system by inhibiting inflammatory cell chemotaxis, cytokine synthesis, adhesion molecule expression, reactive oxygen species production and intracellular signaling pathways (Kanoh and Rubin, 2010). A study conducted by Kraft et al. (2002) focused on the effect of clarithromycin on lung function of *M. pneumoniae* infected and uninfected asthmatic patients. They determined clarithromycin treatment caused improvement in forced expiratory volume and reduced airway expression of IL-5, but only in *M. pneumoniae* positive patients. These findings suggest, in cases of *M. pneumoniae*, macrolides may act primarily as antibiotics as well as an anti-inflammatory agent. The optimal dosage and duration of therapy is not clear; however, 10–14 days is generally recommended. The use of steroids in combination with macrolides has also been recommended in severe cases of *M. pneumoniae* pneumonia.

Macrolide-resistant *M. pneumoniae* was first reported in Japan in Okazaki et al. (2001) and has since been continuously reported in increasing percentages among the population (Matsuoka et al., 2004; Miyashita et al., 2012, 2013; Kawai et al., 2013; Hanada et al., 2014). However, the prevalence of macrolide-resistant *M. pneumoniae* varies among countries. Cao et al. (2010), China documented a 69% prevalence of resistance as well as treatment failure, but no cases of macrolide-resistant *M. pneumoniae* were found from 1997 to 2008 in the Netherlands (Spuesens et al., 2012). It is well-established, point mutations, leading to A-to-G transitions in the peptidyl transferase loop domain V of the 23S rRNA gene at positions 2063 and 2064, reduce the affinity of macrolide for the ribosome (Suzuki et al., 2006; Dumke et al., 2010a). Identification of these resistant strains currently relies on restriction fragment length polymorphism or gene sequence analysis. The creation of a laboratory technique, such as qRT-PCR to rapidly detect macrolide resistant strains may be useful for surveillance and outbreak situation.

Of the major bacterial respiratory pathogens including *Streptococcus pneumoniae* and *Haemophilus influenzae*, *M. pneumoniae* is the only one without an available vaccine (Nir-Paz et al., 2012). During the 1960 and 1970s a number of studies were carried out testing immunogenicity and the protective efficacy of several different vaccines. A recent meta-analysis by Linchevski et al. (2009), has shown the overall pneumonia prevention efficacy of those studies was 41% (54% for *M. pneumoniae* specific pneumonia), when diagnosis was based on culture or serology. No serious adverse effects were reported and only mild local reactions were suffered.

Schurwanz et al. (2009), P1 and P30 adhesions showed strong reactivity with human and animal sera, proving as promising candidates for further vaccine formulation and optimization. However, in 2011 study reported vaccination with only an avirulent P30 mutant resulted in disease exacerbation in mice (Szczepanek et al., 2012). Most recently though the subcutaneous administration of a P1–P30 chimeric recombinant protein, followed by two intranasal booster administrations induced high, consistent, and long lasting IgA levels in guinea pigs (Hausner et al., 2013). The CARDS toxin could also serve as an effective vaccine candidate (Kannan et al., 2011).

*Mycoplasma pneumoniae* is increasingly the cause of upper and lower respiratory tract infections for adults and children. It is associated with prolonged carriage and lacks natural protective immunity following primary infections. Continued development of a vaccine for high-risk individuals such as school children, military recruits, and elderly people in nursing homes or long term hospital care, may help to reduce morbidity from pneumonia and secondary complications. A vaccine may also slow the development of other macrolide resistant strains and reduce the impact of macrolide-resistant strains in communities and epidemics.

## Conclusion

*Mycoplasma pneumoniae* is a commonly found pathogen within adults around the world. It is a common cause of pneumonia but can also initiate other extrapulmonary manifestations. The simplicity of the genome and the small size of mycoplasmas have led many to conclude these organisms are uncomplicated. In reality, much regarding the microbiology and pathogenesis of this organism remains unknown. A variety of respiratory diseases portray similar clinical symptoms. Recent and continued advances in multiplex PCR and qRT-PCR may prove useful in distinguishing the pathogen causing disease. In many situations, the mild symptoms of *M. pneumoniae* pneumonia may be ignored by the patient and remain undiagnosed.

Much energy has been devoted to the CARDS toxin. Information regarding its structure and involvement in pathogenesis may prove useful to the continued development of more effective options for both diagnostics and therapies. Currently, there is limited availability of rapid and accurate testing methods for *M. pneumoniae.* Further development of rapid tests specifically to distinguish macrolide-resistant strains may also be useful within Asia. Physicians and patients around the world must continue to monitor macrolide-resistance strains, preventing dissemination and increased incidence. However, the development of an effective vaccine could prove useful at reducing the burden among the elderly and within the workforce around the world.

## Author Contributions

GP and TK made substantial contributions to the conception and design of the work. GP primarily drafted the work and TK and JF revised it critically for important intellectual content. All had final approval of the version to be published and agree to be accountable for all aspects of the work in ensuring that questions related to the accuracy or integrity of any part of the work are appropriately investigated and resolved.

## Conflict of Interest Statement

The authors declare that the research was conducted in the absence of any commercial or financial relationships that could be construed as a potential conflict of interest.
